# Developmental Nuclear Localization and Quantification of GFP-Tagged EB1c in *Arabidopsis* Root Using Light-Sheet Microscopy

**DOI:** 10.3389/fpls.2015.01187

**Published:** 2016-01-05

**Authors:** Dominik Novák, Anna Kuchařová, Miroslav Ovečka, George Komis, Jozef Šamaj

**Affiliations:** Department of Cell Biology, Centre of the Region Haná for Biotechnological and Agricultural Research, Palacký University OlomoucOlomouc, Czech Republic

**Keywords:** end-binding 1c (EB1c), nucleus, root apex, development, transition zone, light-sheet microscopy

## Abstract

The development of the root apex is determined by progress of cells from the meristematic region to the successive post-mitotic developmental zones for transition, cell elongation and final cell differentiation. We addressed root development, tissue architecture and root developmental zonation by means of light-sheet microscopic imaging of *Arabidopsis thaliana* seedlings expressing END BINDING protein 1c (EB1c) fused to green fluorescent protein (GFP) under control of native *EB1c* promoter. Unlike the other two members of the EB1 family, plant-specific EB1c shows prominent nuclear localization in non-dividing cells in all developmental zones of the root apex. The nuclear localization of EB1c was previously mentioned solely in meristematic cells, but not further addressed. With the help of advanced light-sheet microscopy, we report quantitative evaluations of developmentally-regulated nuclear levels of the EB1c protein tagged with GFP relatively to the nuclear size in diverse root tissues (epidermis, cortex, and endodermis) and root developmental zones (meristem, transition, and elongation zones). Our results demonstrate a high potential of light-sheet microscopy for 4D live imaging of fluorescently-labeled nuclei in complex samples such as developing roots, showing capacity to quantify parameters at deeper cell layers (e.g., endodermis) with minimal aberrations. The data presented herein further signify the unique role of developmental cell reprogramming in the transition from cell proliferation to cell differentiation in developing root apex.

## Introduction

Root development in higher plants is early defined in the developing embryo as soon as root meristem initials appear (Scheres and Berleth, 1998). Thereon, the further growth of the primary root as exemplified in the model dicot *Arabidopsis thaliana* progresses through formative periclinal and proliferative anticlinal divisions in the root meristem and through post-mitotic cell elongation. In this way the root can be anatomically defined laterally by the existence of distinct cell files and longitudinally by the formation of distinct root zones.

In a center wise fashion, root cell files can be discerned to the central cylinder formed by protoxylem and protophloem, surrounded by the pericycle, and followed by the endodermis, the cortex and finally the epidermis that forms the outer root layer. All different root cell types strictly originate from stem cells surrounding the quiescent center at the very root tip (Weigel and Jurgens, 2002). During the growth of the root apex, cells within certain cell files progress through different growth stages in a highly regulated manner. They undergo proliferative anticlinal divisions which are followed by elongation and finally by terminal differentiation in a relatively short time period. In this respect, the root apex is longitudinally divided into four distinguishable zones—meristematic, transition, elongation, and differentiation (Verbelen et al., 2006; Baluška and Mancuso, 2013). The meristematic zone, is characterized by successive cell divisions of non- or minimally elongating cells (van der Weele et al., 2003). In the elongation zone, cell length increases and cell divisions are suppressed. In many classical anatomical studies, the boundary region between meristematic and elongation zone is often neglected. Nevertheless, previous studies demonstrated a population of nearly isodiametric cells within all cell files, with particular characteristics in intracellular architecture such as actin organization (Baluska et al., 1997) or cellular functions such as fluid phase endocytosis (Samaj et al., 2004). This cell population forms a distinct post-mitotic zone in dicots and monocots and it is called transition zone. The transition zone (or otherwise called distal elongation zone; DEZ; Baluška et al., 1990; Ishikawa and Evans, 1993) is considered to form an important link between the meristematic and elongation zone.

The transition zone is interpolated between the meristematic and the elongation zone while cells in this zone are also polarized. The transition zone is sensitive to a variety of stimuli, including plant hormones, effects of cytoskeletal disrupting drugs, gravity, light, oxygen or heavy metal exposure (Illés et al., 2006; Dello Ioio et al., 2007, 2008; Ruzicka et al., 2009; Baluška and Mancuso, 2013; Eleftheriou et al., 2015). Moreover, cells of the root transition zone exhibit unique physiological behaviors including oscillations of ion and hormone fluxes and also of gene expression (Benková and Hejatko, 2009; McLamore et al., 2010; Baluška and Mancuso, 2013).

During cell remodeling in the elongating root, nucleus shape and position undergo dramatic changes (Chytilova et al., 2000; Ketelaar et al., 2002; Sliwinska et al., 2012). While cell expansion proceeds in the elongation zone, DNA amount (C value) increases by switching from mitotic to endoreduplication cycles (Hayashi et al., 2013). The switch from the mitosis to endoreduplication is accompanied by suppression of mitotic entry by inactivation of mitotic cyclin-dependent kinases (Adachi et al., 2011). Endoreduplication causes nuclear enlargement and reshaping.

Microtubule plus-end-tracking proteins are mostly conserved proteins throughout eukaryotes (Jiang and Akhmanova, 2011). They regulate MT plus-end dynamics (Bisgrove et al., 2004; Hamada, 2007; Akhmanova and Steinmetz, 2008). Proteins from End-binding 1 (EB1) family were first identified as +TIPs in plants and are highly conserved both evolutionary and structurally throughout kingdoms (Chan et al., 2003; Mathur et al., 2003; Bisgrove et al., 2008). Significant influence of the microtubule plus-end dynamics refers them as key players in the cell expansion and cell division (Tirnauer et al., 2002a,b; Draviam et al., 2006; Akhmanova and Steinmetz, 2008). They are composed of N-terminal microtubule-interacting calponin-homology (CH) domain and C-terminal EB1 unique homology domain which mediates EB1 protein dimerization and interaction with other proteins (Komarova et al., 2009). The *Arabidopsis* genome encodes for three EB1 isoforms (Bisgrove et al., 2008), EB1a (At3g47690), EB1b (At5g62500), and EB1c (At5g67270). EB1a and EB1b share 78% aminoacid identity and they are typical members of microtubule plus-end-tracking proteins. Fused with GFP protein, EB1a and EB1b distinctly decorate the plus end of cortical and mitotic microtubules (Chan et al., 2003; Mathur et al., 2003). In contrast, EB1c shows different localization and seems to have different functions (Bisgrove et al., 2008; Komaki et al., 2010). It shares only 49% aminoacid identity with EB1a and EB1b and it is considered to be plant-specific (Bisgrove et al., 2008). Its aminoacid sequence differs remarkably at its C-terminal part. EB1a and b possess acidic aminoacid residues in the C terminal region, while C terminal part of EB1c contains basic residues encompassing a nuclear localization sequence. This is consistent with the nuclear localization of EB1c in interphase and post-cytokinetic cells (Komaki et al., 2010). Furthermore, the primary sequence of EB1c, unlike EB1a and EB1b, contains five unique SP motifs and one D-domain motif suggesting its targeting by protein kinases implicated in the cell cycle including cyclin-dependent kinases and mitogen-activated protein kinases (MAPKs) (Samajova et al., 2013). In animal kingdom, proteins from EB1 family were shown to be regulated by phosphorylation (Tamura and Draviam, 2012). A putative interaction between EB1c and MAPKs was recently shown also in plants (Kohoutová et al., 2015). All plant EB1 proteins decorate mitotic microtubules (Chan et al., 2003; Dixit et al., 2006), however, solely EB1c becomes actively transported to the nucleus at the end of cytokinesis (Bisgrove et al., 2008; Komaki et al., 2010). Single *eb1c* mutants showed collapsed spindles and fragmented phragmoplasts (Komaki et al., 2010) and they exhibited hypersensitivity to microtubule-disrupting drugs (Bisgrove et al., 2008).

Since EB1 proteins are typical microtubule plus-end-tracking proteins, all previous studies were focused on the possible function of EB1c during the cell division where EB1c decorates mitotic microtubules (Bisgrove et al., 2008; Komaki et al., 2010; Ho et al., 2011). However, accumulation of EB1c in the mitotic interphase and post-mitotic nuclei was undermined. Consequently, we extend the localization studies of EB1c protein to demonstrate its occurrence and accumulation in the nuclei of non-dividing mitotic cells but especially in cells of post-mitotic transition and elongation root zones. Moreover, we present the first quantitative study of EB1c nuclear content in non-dividing and post-mitotic cells of different root tissues at different developmental root zones with special emphasis on transition zone.

Most of the studies which dissected root anatomy and its developmental establishment so far were based on roots growing on the interface of solidified nutrient media under conditions of uniform seedling illumination (e.g., Ruzicka et al., 2009; Sliwinska et al., 2012) and only few studies elaborated root architecture and subcellular organization of soil grown roots (e.g., Panteris et al., 2013). Moreover, the microscopic elucidation of root cellular and tissue architecture was vastly established with microscopes equipped with horizontal working stages and spherical aberration-limited working distances (Petricka et al., 2012). Therefore, experimental conditions which documented root development in the laboratory setup have been largely deviating from the natural environment. For this reason we employed light-sheet microscopy as an excellent tool for near-physiological, live cell imaging in long-term with the potential to study dynamic developmental processes using firmly established protocol for sample preparation and imaging (Maizel et al., 2011; Sena et al., 2011; Ovecka et al., 2015). With the setup of the light-sheet microscope used herein it is possible: (a) to mount seedlings vertically allowing the root to grow along the gravity vector, (b) to achieve minimal illumination of the root as happens under natural conditions, and (c) to grow root embedded in solid medium closely resembling conditions in the soil.

From the technical point of view, the light-sheet microscope allows minimal exposure of roots carrying GFP-tagged proteins to phototoxic irradiation while importantly provides the means for fast and adequate resolution in the three spatial dimensions (Maizel et al., 2011; Ovecka et al., 2015). This is made possible by the low excitation laser powers and the relatively high numerical aperture objectives used for excitation of the sample and acquisition of the image (Ovecka et al., 2015).

The purpose of the present study is to follow the developmental correlation between nuclear levels of EB1c and the organization of the root of *Arabidopsis thaliana* in distinct zones and cell files. We did so in living plants which were imaged under minimally invasive light-sheet microscopy. Thus, we were able to address and quantify nuclear localization of EB1c in meristematic, transition and elongation zones and we extend the observations to three important root tissue layers in all zones: the epidermis, the cortex and the endodermis, correlating nuclear size with EB1c expression levels.

## Materials and methods

### Molecular methods

Genomic DNA isolated from fresh leaves of 7-days-old *Arabidopsis thaliana* (L.) Heynh. (ecotype Columbia) seedlings was used for amplification of the complete coding region of *EB1c* gene together with its upstream promoter region. *EB1c* promoter region was identified according to previously published data (Komaki et al., 2010) and designed using *Arabidopsis* Sequence Viewer (http://arabidopsis.org). The promoter sequence *pEB1c* was suggested to comprise of 663 base pairs upstream of the start codon of the *EB1c* gene. For the cloning, we used Gateway® technology. Primers were designed according to manufacturer's instructions. Whole genomic fragment was recombined into pDONR207 Gateway® vector and subsequently transferred by LR recombination reaction into the Gateway® destination vector pGWB450 designed for C-terminal GFP fusion with kanamycin resistance for plant selection. All recombinations were confirmed by sequencing. Expression vector pGWB450::pEB1c::EB1c:GFP was transformed into *Agrobacterium tumefaciens*, strain GV3101. Transformed Agrobacteria were used for several independent transformations of *Arabidopsis* plants (Clough and Bent, 1998; Davis et al., 2009). Seedlings were selected on kanamycin (50 mg/ml) selection medium to identify T1 transgenic plants. T1 transformants carrying pEB1c::EB1c:GFP constructs were checked for phenotype in comparison to control plants. No phenotypes were discerned and thus the T2 generation of *Arabidopsis* plants expressing pGWB450::pEB1c::EB1c:GFP was harvested and used for further experiments.

### Protein extraction, SDS-PAGE and immunoblotting

Twelve-days old *Arabidopsis* plants expressing EB1c-GFP were analyzed on fluorescent stereomicroscope Leica MZ FLIII (Leica Microsystems, Germany) for EB1c-GFP signal. Protein extraction was carried out from roots of plants expressing EB1c-GFP and from wild type *Arabidopsis thaliana* (L.) Heynh. (ecotype Columbia). Roots were harvested, weighted, flesh frozen and immediately ground in the liquid nitrogen. Powder was extracted in extraction buffer (1:1, w/v) (50 mM Na-HEPES pH 7.5, 150 mM NaCl, 1 mM MgCl_2_.6H_2_O, 1 mM EGTA, 1 mM DTT, 1 mM NaF) supplemented with protease inhibitors Complete (Roche, Germany) and phosphatase inhibitors PhosStop (Roche, Germany). Crude extract was centrifuged 10 min, 8000 g at 4°C. Resulting supernatant was used for SDS-PAGE and subsequent western blot analysis. 15 μg of total protein was loaded on 8% SDS-PAGE gels followed by immunoblotting with PVDF membrane and Western blotted with antibody against GFP (anti GFP rabbit ABCAM AB290) in dilution of 1:2000. Secondary antibody (goat anti-rabbit, Santa Cruz Biotechnology) was used in dilution of 1:5000. After incubation in ECL reagents (according to manufacturer instructions), immunoreactive bands were documented using the BioRad ChemiDoc™ MP System.

### Plant material and sample preparation for light-sheet imaging

Seeds of *Arabidopsis thaliana* (L.) Heynh. (ecotype Columbia) transgenic line expressing *pEB1::cEB1c:GFP* were surface sterilized, plated onto solidified MS medium and kept in 4°C for 4 days. After this period seeds were transferred to round 90 × 25 mm Petri dishes filled with 80 ml of ½MS medium solidified with 0.6% w/v Phytagel, and placed into small depressions facilitating gravitropic root growth inside solidified culture medium. Plates were cultivated in culture chamber horizontally for 2 days at 22°C, 50% humidity, 16/8-h light/dark cycle. After germination of seedlings when they were 1-days-old, they were enclosed by fluorinated ethylene propylene (FEP) tube with an inner diameter of 1.1 mm and wall thickness of 0.2 mm (Wolf-Technik, Germany). FEP tubes were carefully inserted into culture medium to enclose individual seedling inside. After 24 h seedlings in FEP tubes were removed from the plate, transferred to the microscope and prepared for imaging according to established protocol (Ovecka et al., 2015). Seedlings in FEP tubes were prepared according to an “open system,” where root is growing in the original Phytagel-solidified culture medium in the lower part of FEP tube and shoot is growing in the upper aerated part of the FEP tube. This approach provides green parts of seedlings with continuous access to oxygen and allows growth and development of roots and shoots in the microscope chamber during long-term imaging experiments (Ovecka et al., 2015). All experiments and measurements were done on 2-days old seedlings.

### Light-sheet microscopy

Developmental live cell imaging was done with the light-sheet Z.1 fluorescence microscope (Carl Zeiss, Germany), equipped with W Plan-Apochromat 20 ×/1.0 NA water immersion detection objective (Carl Zeiss, Germany) and LSFM 10x/0.2 NA illumination objective (Carl Zeiss, Germany). Seedlings were imaged using dual-side illumination by a light-sheet modulated into a pivot scan mode, with excitation laser line 488 nm and with emission filter BP505-545. Image acquisition was done every 2 min in Z-stack mode for a time period of 2–5 h. Scaling of images in x, y, and z dimensions was 0.228 × 0.228 × 0.477 μm. To prevent the movement of the growing root apex out of the field of view, images were acquired in two subsequent views coordinated to each other in y coordinate. Images were recorded with the PCO.Edge sCMOS camera (PCO AG, Germany) with the exposure time 25 ms.

### Measurements, statistical analyses, and *in silico* predictions

From images of the whole root acquired using Zen 2014 software (Carl Zeiss, Germany) subsets of data were created, with defined x−, y−, and z− dimensions comprising whole volume of several nuclei from one particular cell file. Several subsets were created in order to segment nuclei of all cells of particular cell file in ordered positions from the stem cells surrounding quiescence center of the root up to visible cell differentiation at the end of elongation zone. All subsets were transformed to 2D images using Maximum intensity projection function of the Zen 2014 software. In all images uniform correction of brightness and contrast was done before they were exported for image analysis. All quantitative data were produced with publicly available software CellProfiler 2.1.1 (http://www.cellprofiler.org; Carpenter et al., 2006; Lamprecht et al., 2007). Nuclear area from 2D images that represent surface projection of the nuclear volume (referred herein as nuclear surface area) was measured as the actual number of pixels in the manually defined region multiplied by the pixel area. Mean intensity values were calculated as the average pixel intensity in the defined region, integrated intensity values were calculated as the total pixel intensity in the defined region. Values were subsequently normalized to a 0–1 range using the following formula: x_N_ = (x_i_-x_min_)/x_max_-x_min_ (where x_N_ = normalized intensity, x_i_ = absolute intensity, x_min_ = minimum absolute intensity and x_max_ = maximum absolute intensity). Thus, all biological variables within measured root tips were brought into the comparable proportions and plant—to—plant differences in the expression of EB1c-GFP were compensated. Data from 4 individual cell files were collected and evaluated separately for epidermis, cortex and endodermis from two independent experiments (two independent roots). Final statistical data evaluation and plot production was done with Microsoft Excel software.

Prediction of putative nuclear export sequences was performed using the NetNES 1.1 server with the accession numbers of the three *Arabidopsis thaliana* EB1 isoforms (EB1a, AT3G47690; EB1b, AT5G62500; and EB1c, AT5G67270) (la Cour et al., 2004). For protein domain structure illustration, DOG 1.0 illustrator was used (Ren et al., 2009).

## Results

**Figure 1:**
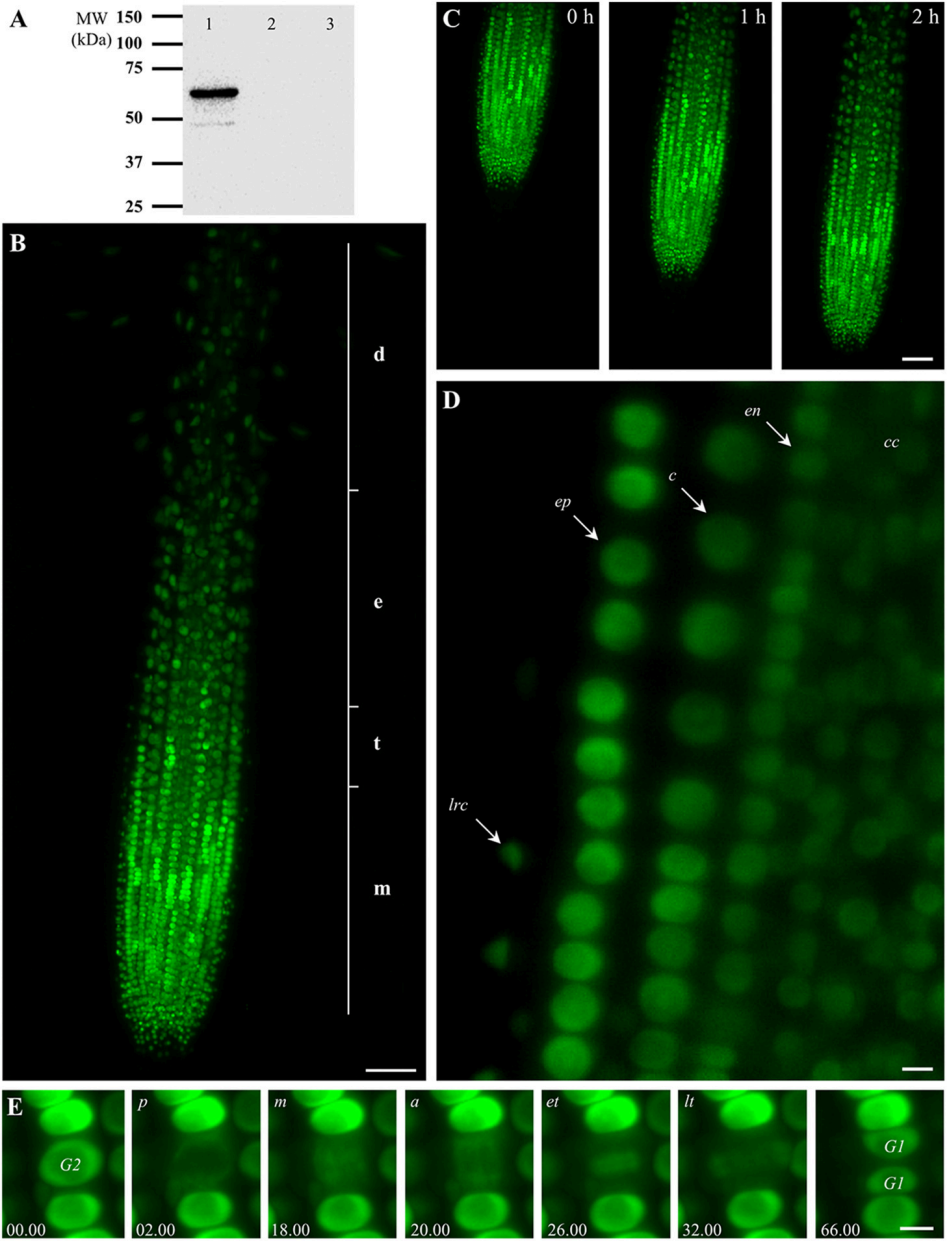
**Characterization of transgenic ***Arabidopsis thaliana*** plants expressing EB1c-GFP driven by EB1c own promoter and localization of EB1c-GFP**. **(A)** SDS-PAGE followed by western blot analysis using anti-GFP antibody from protein extract of *A. thaliana* T2 plants expressing EB1c-GFP (lane 1), protein extract from untransformed Col-0 plants (lane 2), and protein extract from *Arabidopsis* plants expressing EB1c-GFP immunoblotted only with secondary antibody (lane 3). **(B)** General overview of the root tip of 2-days old *A. thaliana* seedling expressing EB1c-GFP. Average root zonation into individual cell developmental zones consisting meristematic zone (m), transition zone (t), elongation zone (e), and differentiation zone (d) is depicted. EB1c-GFP was localized in nuclei of root cells, with particularly strong expression level in cells of the root meristematic zone. **(C)** Live cell imaging of seedling growing inside of the light-sheet microscope over the period of 2 h. **(D)** Localization of EB1c-GFP by light-sheet microscopy in cells of individual root tissue layers, in epidermis (arrow, ep), cortex (arrow, c), endodermis (arrow, en), central cylinder (arrow, cc), and lateral root cap cells (arrow, lrc). In all imaged tissues, light-sheet microscopy revealed clearly nuclear localization of EB1c-GFP in root cells. **(E)** Localization of EB1c-GFP by light-sheet microscopy during mitotic cell division of root cells in the meristematic zone. EB1c-GFP relocated from G2 stage nuclei to spindle in prophase (p), metaphase (m), and anaphase (a), to phragmoplast at early and late telophase (et, lt), and redistributed back to reconstructed G1 stage nuclei after cell division. Time progression of the cell division is indicated in min. Scale bar = 50 μm in **(B,C)** and 5 μm in **(D,E)**.

We studied the *in vivo* subcellular nuclear localization of the EB1c protein during root development in stably transformed *Arabidopsis thaliana* plants. Beside the already published presence of nuclear localization sequence in EB1c protein sequence (Komaki et al., 2010), our *in silico* search using NetNES 1.1 prediction server (la Cour et al., 2004) revealed the occurrence of putative nuclear export signals at positions 213L, 215I, 217S, and 218L for EB1c (Supplementary Figure 1). At the same respect, we compared the other EB1 family members in *Arabidopsis*, EB1a and EB1b. NES was not predicted for EB1b, while for EB1a, there is one prediction at position 193I albeit with a low score over the threshold. We prepared *EB1c:GFP* construct driven by its own promoter. The 663 bp long promoter sequence and the complete coding region of *EB1c* gene were cloned using Gateway® technology into the binary vector and subsequently transformed into *A. thaliana* plants (ecotype Col-0) while T2 generation of plants was used for experiments. To prove the expression of EB1c-GFP fusion protein in the plants, we performed SDS-PAGE with subsequent Western-blot analysis using seedlings expressing EB1c-GFP and Col-0 as a negative control (Figure 1A). EB1c-GFP protein signal was clearly detected using anti-GFP antibody at the molecular mass corresponding to 64 kDa which is the predicted size of the fusion protein. As negative controls, we used extracts from untransformed wild-type plants as well as extracts from plants expressing EB1c-GFP treated only with secondary antibody (Figure 1A).

Root growth and development require passage of root cells through successive developmental zones. Large extend of this process from spatial and temporal point of view requires special microscopic applications for effective live cell imaging. Developmental light-sheet microscopy overcomes these limitations and allows real-time or time-lapse imaging of whole developing seedlings (Ovecka et al., 2015). We performed live cell imaging with seedlings growing over a period between 2 and 5 h inside the light-sheet microscope. EB1c-GFP was localized in nuclei of all non-dividing root cells within the root apex, with particularly strong expression level in cells of the root meristematic zone (Figure 1B). General overview of EB1c-GFP expressing roots revealed zonation of the root apex into different cell developmental zones, namely into meristematic, transition, elongation, and differentiation zones (Figure 1B). Seedlings were prepared and cultivated in cylinders of Phytagel-solidified culture medium. During imaging over a range of several hours, seedlings exhibited undisturbed continuous root growth inside of the microscope (Figure 1C, Supplementary Movie 1). Average root growth rate of 2-days old seedlings expressing EB1c-GFP in the light-sheet microscope was 1.686 (± 0.721) μm.min^−1^ (±SD, *n* = 6).

Light-sheet microscopy, in addition to time-lapse imaging of the entire root development, allowed localization of EB1c-GFP at the cellular and subcellular levels. At the level of cellular resolution, this method was suitable for visualization not only surface cells and tissues of the root, like lateral root cap cells and epidermis, but further allowed visualization of individual cells from inner tissues of the *Arabidopsis* root including the cortex, the endodermis and the central cylinder. In all imaged tissues, light-sheet microscopy revealed clearly nuclear localization of EB1c-GFP in root cells (Figure 1D). Subcellular resolution of the light-sheet microscopy was documented during mitotic cell division of root cells in the meristematic zone, where EB1c-GFP relocated from G2 stage nuclei to mitotic spindles and cytokinetic phragmoplasts during the respective cell division stages and finally was redistributed back to reconstituted G1 stage nuclei after completing cell division (Figure 1E).

**Figure 2:**
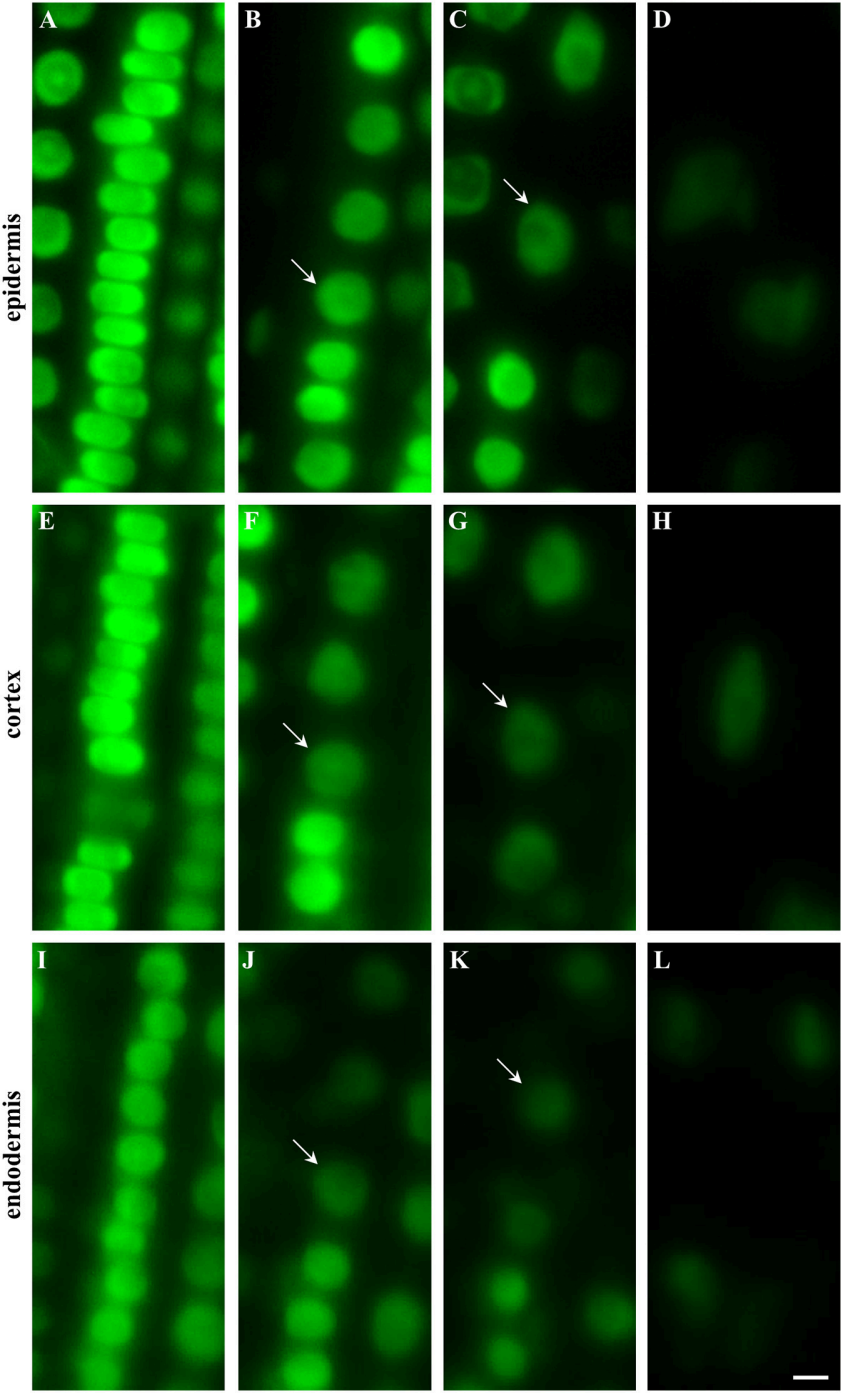
**Shoot-ward developmental zonation in the root apex of transgenic ***Arabidopsis thaliana*** plants expressing EB1c-GFP protein**. Cell arrangement and position were assessed in individual cell files of epidermis **(A–D)**, cortex **(E–H)** and endodermis **(I–L)**. Typical appearance of cells in each tissue layer with nuclear localization of EB1c-GFP is shown for meristematic zone **(A,E,I)**, at the meristem-transition zone border **(B,F,J)**, at the transition zone-elongation zone border **(C,G,K)** and within the elongation zone **(D,H,L)**. Arrows define nuclei of first cell within the transition zone **(B,F,J)** and first cell within the elongation zone **(C,G,K)**. Fluorescence intensity of images is presented to the scale recorded during the acquisition of each individual cell type. Scale bar = 5 μm.

The longitudinal developmental zonation of the root apex of plants expressing EB1c-GFP fusion protein into different developmental zones was determined in individual cell files of epidermis (Figures 2A–D, arrows in B and C denote the first cell of each consecutive zone), cortex (Figures 2E–H, arrows in F and G denote the first cell of each consecutive zone) and endodermis (Figures 2I–L, arrows in J and K denote the first cell of each consecutive zone). The cell arrangement in the meristematic zone of the epidermis was influenced by frequent cell divisions rather than by cell elongation (along longitudinal root axis). This leads to generation of tightly-packed wide but very short cells, with compressed nuclei (Figure 2A). Very similar cell and nuclear shapes were observed within the meristematic zone in the cortex layer (Figure 2E). In the endodermis nuclei of cells in the meristematic zone appeared smaller and due to different ratio between cell width and cell height their shape was not deformed to the same extent as in the epidermis and the cortex (c.f. Figures 2A,E,I). With termination of the mitotic activity in the meristematic zone, cell sizes, and shapes were changing. At the end of the meristematic zone, nuclei became round and larger in all three layers (Figures 2B,F,J). Before starting rapid cell elongation, however, there was a population of cells with short length, as indicated by short and similar distance between nuclei of individual cells (Figures 2B,F,J). In addition, they showed reduction in the EB1c-GFP fluorescence intensity. Based on these characteristics, an onset of the transition zone placed before rapid cell elongation could be identified in each cell file of epidermis (Figure 2B), cortex (Figure 2F), and endodermis (Figure 2J). Cells from the transition zone of each tissue layer entered subsequently cell elongation zone, which was indicated by further changes in the nucleus size, EB1c-GFP fluorescence intensity and apparent dilatation of distances between neighbor nuclei within the cell files (Figures 2C,G,K). Rapid cell elongation was connected with apparent enlargement of nuclei in epidermis (Figure 2D) and cortex (Figure 2H), changes in nuclear shape in all three tissue layers (Figures 2D,H,L) and further reduction in EB1c-GFP fluorescence intensity.

To characterize the distribution of EB1c-GFP in the apex of *Arabidopsis* root in detail, we quantified the intensity of EB1c-GFP nuclear fluorescence in the previously defined root cell developmental zones (meristem, transition zone, elongation zone and differentiation zone). The quantitative evaluation of the EB1c-GFP protein content in interphase nuclei of root cells was performed in light-sheet images acquired in 4D modes (encompassing x, y, z, and t dimensions). In individual cell files of the epidermis, the cortex and the endodermis all interphase nuclei in order were taken into account, starting from the stem cell niche region and progressing up to cell elongation before cells reached the zone of cell differentiation (i.e., as evidenced by root hair emergence in the root epidermis). In each cell file, the meristem—to—transition zone border (Figures 2B,F,J) and the transition zone—to—elongation zone border (Figures 2C,G,K) were identified. Parameters for identification of particular borders and range of individual cell developmental zones included the spatial extent of the cell division, size, and fluorescence intensity reference values from nuclei of cells in G1 and G2 stages, increase in the nuclear size after termination of the cell division, and increase in the distance between nuclei of individual cells in the elongation zone. Thus, all cells in each cell file were topologically divided into meristematic zone, transition zone, and elongation zone (Figure 1B).

**Figure 3:**
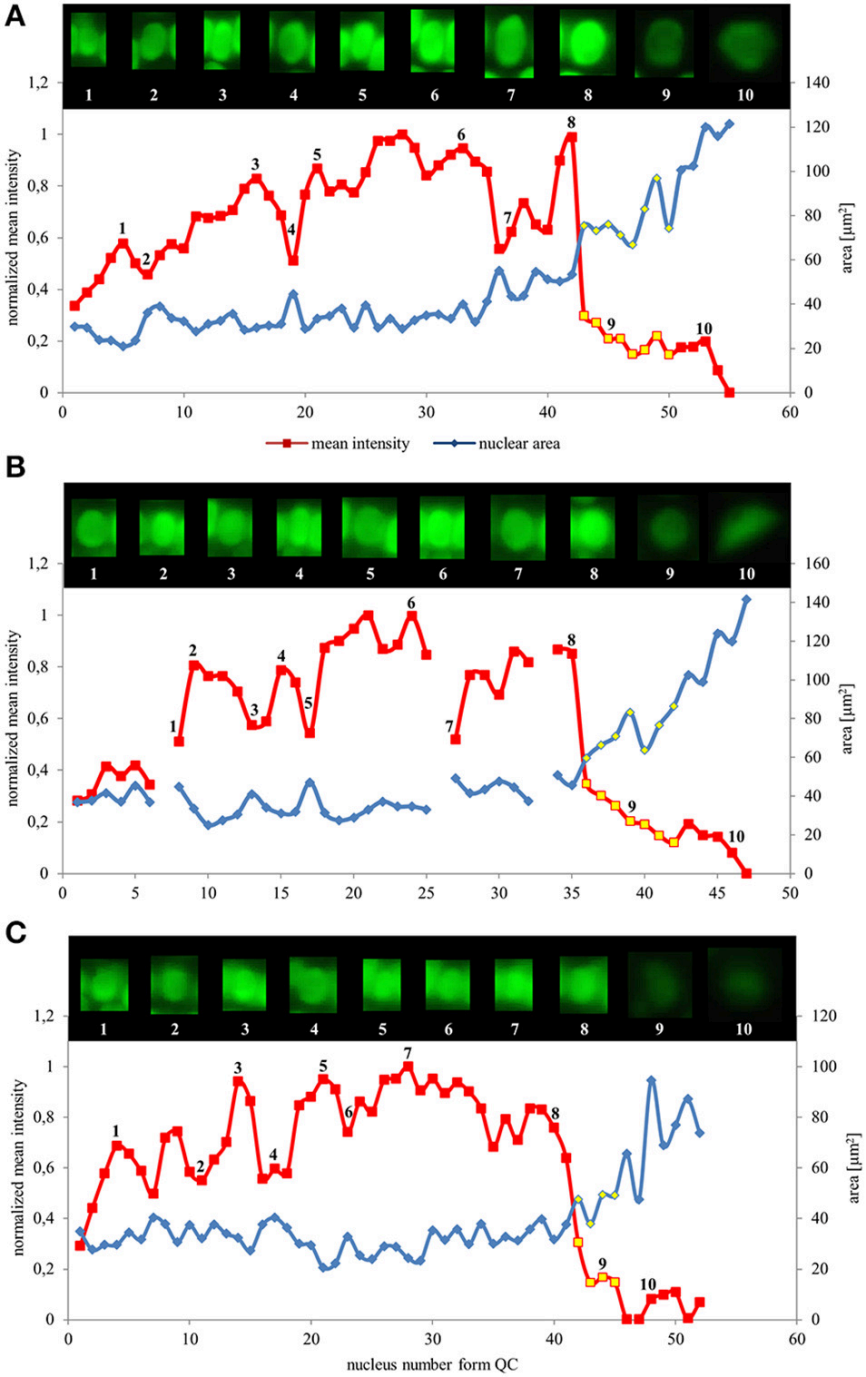
**Nuclear surface area and nuclear EB1c-GFP mean signal intensity distribution in root developmental zones of the root apex in individual cell files**. Relationship between nuclear area (blue line) and nuclear EB1c-GFP mean signal intensity (red line) in respect to cell position counted from the stem cells surrounding quiescent center in individual cell file of epidermis **(A)**, cortex **(B)**, and endodermis **(C)**. Values for nuclei in the transition zone are highlighted by yellow points, which allow distinguishing also meristematic zone (appearing before the transition zone) and elongation zone (appearing after the transition zone). Inset images over the lines show individual nuclei of cells at the actual position, documenting changes in nuclear shape, size, and mean EB1c-GFP fluorescence intensity. Major vertical axis (on the left) represents the values for the normalized mean intensity and minor vertical axis (on the right) represents values for area measurements. Horizontal axis represents the actual cell position counted from the stem cells surrounding quiescent center. Data are shown for one representative cell file from each tissue layer. Interruptions of the curve in the cortex **(B)** are caused by presence of dividing cells within the file.

Values of nuclear surface area and EB1c-GFP mean signal intensity for quantitative evaluation were plotted against cell position counted from the stem cells surrounding quiescence center. Both parameters were measured and evaluated separately for the epidermis, the cortex and the endodermis. We found that in all tissues, as cells proceeded from proliferation to the differentiation, the nuclear surface area increased while fluorescence intensity of EB1c-GFP signal decreased. This trend was apparent from quantification of individual cell files of the epidermis (Figure 3A), the cortex (Figure 3B) and the endodermis (Figure 3C). In the meristematic zone, actively dividing cells contained the smallest nuclei exhibiting the highest EB1c-GFP content. In the transition zone, the mean nuclear fluorescence intensity of EB1c-GFP was steeply decreased. This decline in EB1c-GFP fluorescence intensity continued in the elongation zone while the nuclear area progressively increased (Figure 3). Cross-correlation of nuclear EB1c-GFP mean signal intensity of some individual nuclei with their size and shape at certain position within the cell file revealed negative correlation between nuclear size and mean EB1c-GFP signal intensity in the meristematic zone (numbered insets in Figures 3A–C). This negative correlation trend was stabilized in the transition zone and the elongation zone of all measured cell files in all evaluated tissue layers (Figure 3) as evidenced by the continuous decrease in EB1c-GFP fluorescence intensity with the progressive increase in nuclear size.

**Figure 4:**
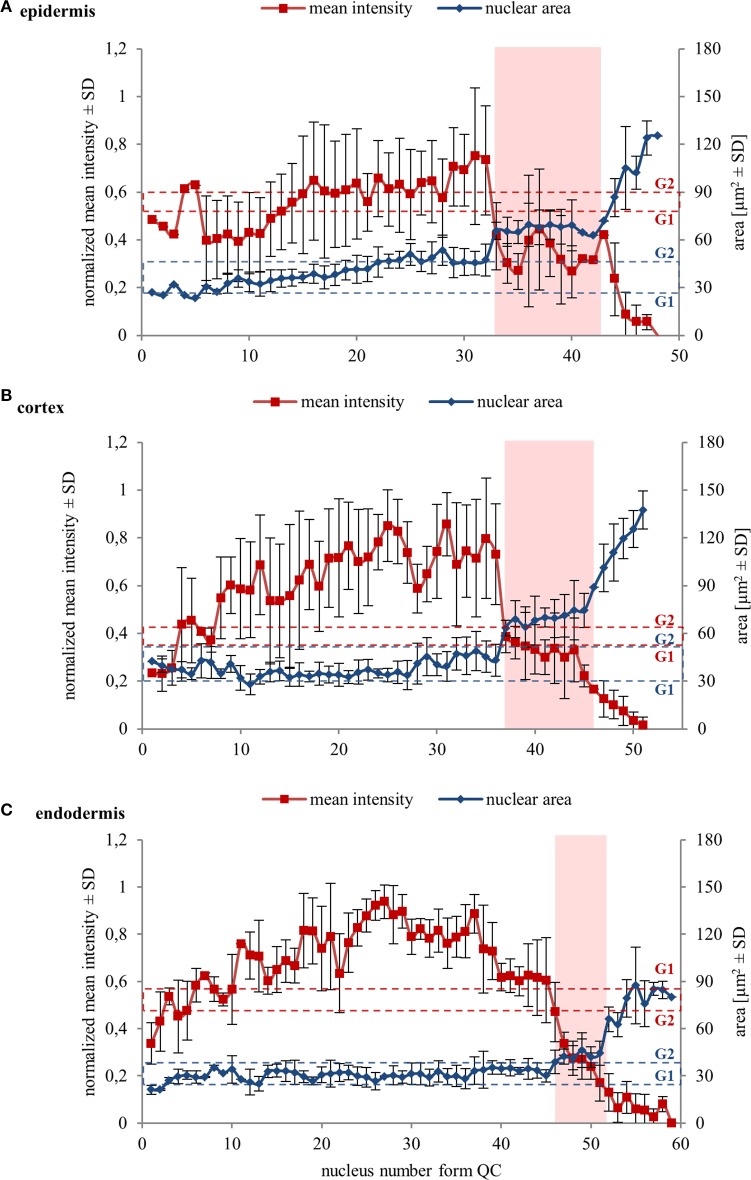
**Nuclear surface area and nuclear EB1c-GFP mean signal intensity in cells of different tissues along diverse developmental root zones**. Average values of nuclear surface area (blue line) and EB1c-GFP mean signal intensity (red line) plotted against cell position counted from the stem cells surrounding quiescence center. Data from 4 individual cell files were collected and evaluated separately for epidermis **(A)**, cortex **(B)**, and endodermis **(C)** from two independent roots. The transition zone is highlighted in pink color. Dashed red lines for average mean intensity and dashed blue lines for average nuclear area of G1 and G2 nuclei are indicated as reference values. Major vertical axis (on the left) represents the values for the normalized mean intensity and minor vertical axis (on the right) represents values for area measurements. Horizontal axis represents the actual cell position counted from the stem cells surrounding quiescence center. Because number of cells in cell files is not the same, data from individual cell files in average graphs were aligned according to their meristem-to-transition zone borders.

Further, we quantified collectively measured data from several individual cell files of two independent roots. Data were evaluated separately for epidermis, cortex, and endodermis. Quantitative evaluation of nuclear surface area values revealed rather stable distribution of this parameter in the meristematic zone of the epidermis. It increased slightly only in meristematic cells at gradually increasing distances from the stem cell region, surpassing slightly even the average reference value for the size of G2 nuclei (Figure 4A). Further recognizable increase in the nucleus size took place within the transition zone, and dramatic increase in the elongation zone of epidermis (Figure 4A). Mean fluorescence intensity of EB1c-GFP in interphase nuclei of epidermis fluctuated considerably; however, it was high in the meristematic cells. In nuclei of cells entering the transition zone mean fluorescence intensity of EB1c-GFP dropped considerably and in nuclei of elongating cells this drop in mean fluorescence intensity was dramatic, reflecting the inversely proportional increase in the nucleus size (Figure 4A).

A similar tendency of stable nuclear size in the meristematic zone, a gradual increase in the transition zone and a considerable increase in the elongation zone was recorded also in cell files of the cortex layer (Figure 4B). Nuclear size in the meristematic zone did not exceed the reference value for the size of G2 nuclei (Figure 4B). Mean fluorescence intensity of EB1c-GFP in interphase nuclei of cortex cells was highest in the meristematic zone. However, it decreased dramatically in the transition zone, keeping further decreasing in the elongation zone as well (Figure 4B).

Size of nuclei of endodermal cells in the meristematic zone was constant, not exceeding the reference value of size measured for G2 nuclei, showing increase only after passage of meristematic cells into the transition zone. Size distribution of nuclei in the transition zone and the elongation zone of endodermis was wider as in cortex and epidermis, but the general tendency of gradual nuclear size increase from meristem through transition zone to elongation zone was maintained also in endodermis (Figure 4C).

Quantitative comparison of nuclear surface area and nuclear EB1c-GFP mean signal intensity in cell developmental zones of the root apex thus showed that the expression of EB1c-GFP decreased along the longitudinal axis of the root apex in all three measured tissue layers. Highest intensity was measured in the meristematic zone where cells are actively dividing, while in the transition zone and further in the elongation zone, expression levels of EB1c-GFP decreased inversely in relation to the nuclear area which progressively increased before entering the differentiation zone.

**Figure 5:**
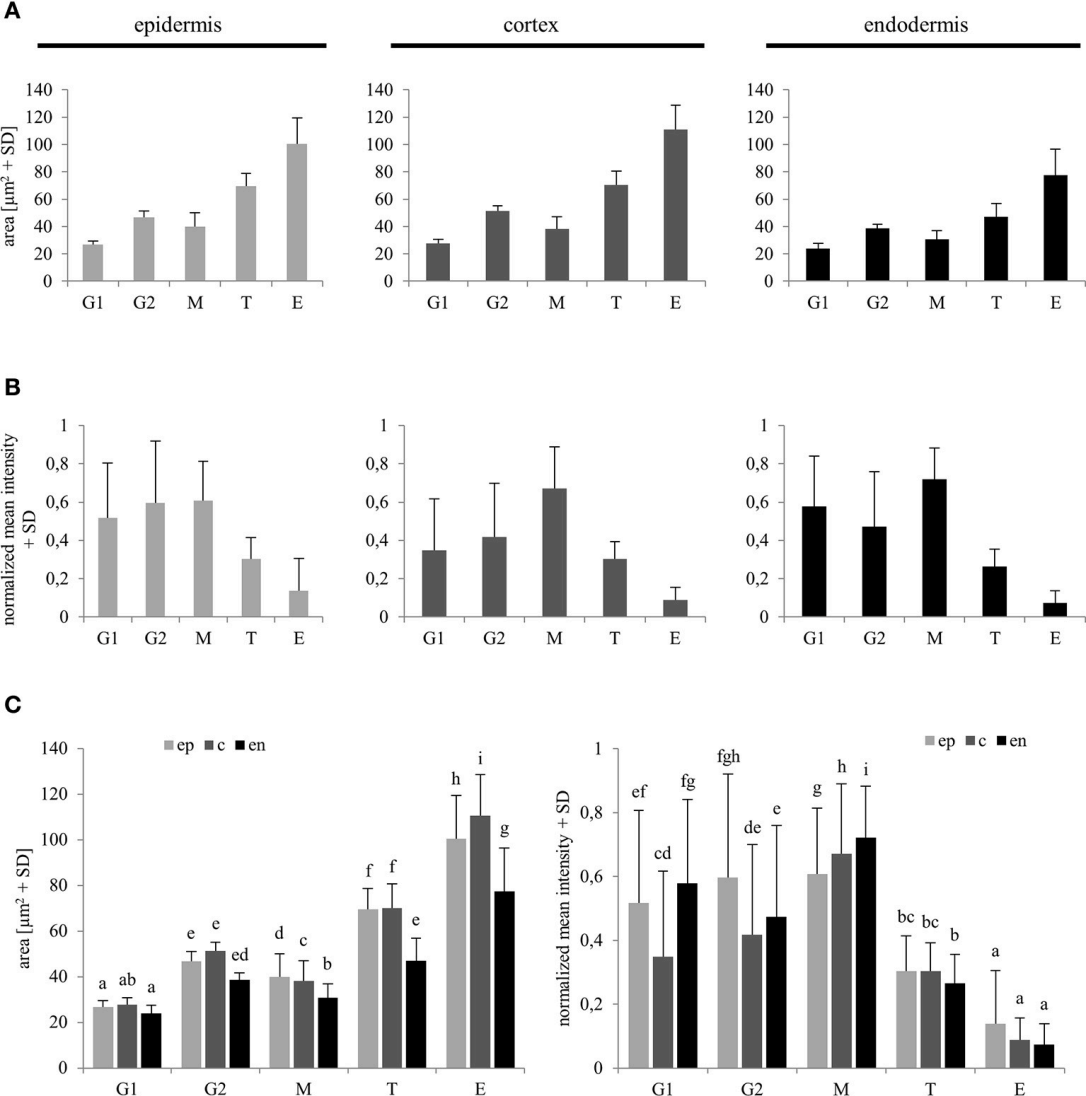
**Reference values of nuclear area and nuclear EB1c-GFP mean signal intensity in G1 and G2 stage meristematic cells as compared to post-meristematic cells**. Average nuclear surface area **(A)** and average nuclear EB1c-GFP mean signal intensity **(B)** in G1 and G2 stage of cell cycle were compared with average values of the same parameters of non-dividing cells from meristem (M), transition zone (T), and elongation (E) zone in epidermis, cortex, and endodermis. Comparison of average nuclear surface area and average nuclear EB1c-GFP mean signal intensity values in individual cell developmental zones among individual tissue layers **(C)**. Different letters represent statistical significance according to One-way ANOVA test at *P* < 0.05.

Reference values for comparison of nuclear surface area and nuclear EB1c-GFP mean signal intensity in all types of measured cells from the root apex were recorded from typical cells of the root meristematic zone, which were present in G1 and G2 stages of mitotic cell division. For safe identification of nuclei in G1 and G2 stages, we took advantage of long-term time-lapse imaging of growing root apex in the light-sheet microscope. Using play-back function we identified and picked cells just before mitotic division and marked them as G2 cells. Daughter cells derived from mitotic division of these cells were marked as G1 cells (Figure 1E). We compared obtained average values of G1 and G2 nuclei with average values of all measured nuclei from non-dividing cells of meristematic zone, and all cells of transition and elongation zones for nuclear surface area (Figure 5A) and nuclear EB1c-GFP mean signal intensity (Figure 5B). This comparison was done separately for epidermis, cortex and endodermis. Average values of nuclear surface area of meristematic cells corresponded well with reference values from G1 and G2 nuclei. Most importantly, the average size of nuclei in the meristematic zone did not exceed the size of G2 nuclei (Figure 5A). Size of nuclei in transition zone as well as in elongation zone was significantly higher. Significant differences in size of nuclei were also observed between transition zone and elongation zone (Figures 5A,C). Similarly, average values of nuclear EB1c-GFP mean signal intensity of meristematic cells corresponded well with reference values from G1 and G2 nuclei, and there were found significantly lower values of nuclear EB1c-GFP mean signal intensity in cells of transition zone and elongation zone in comparison to cells of meristematic zone and reference G1 and G2 nuclei (Figures 5B,C). From these data we can conclude that both nuclear size and EB1c-GFP fluorescence intensity showed significant differences between cells of meristematic zone, transition zone and elongation zone, and supported the correct classification of cells of the root apex into three distinct developmental zones.

**Figure 6:**
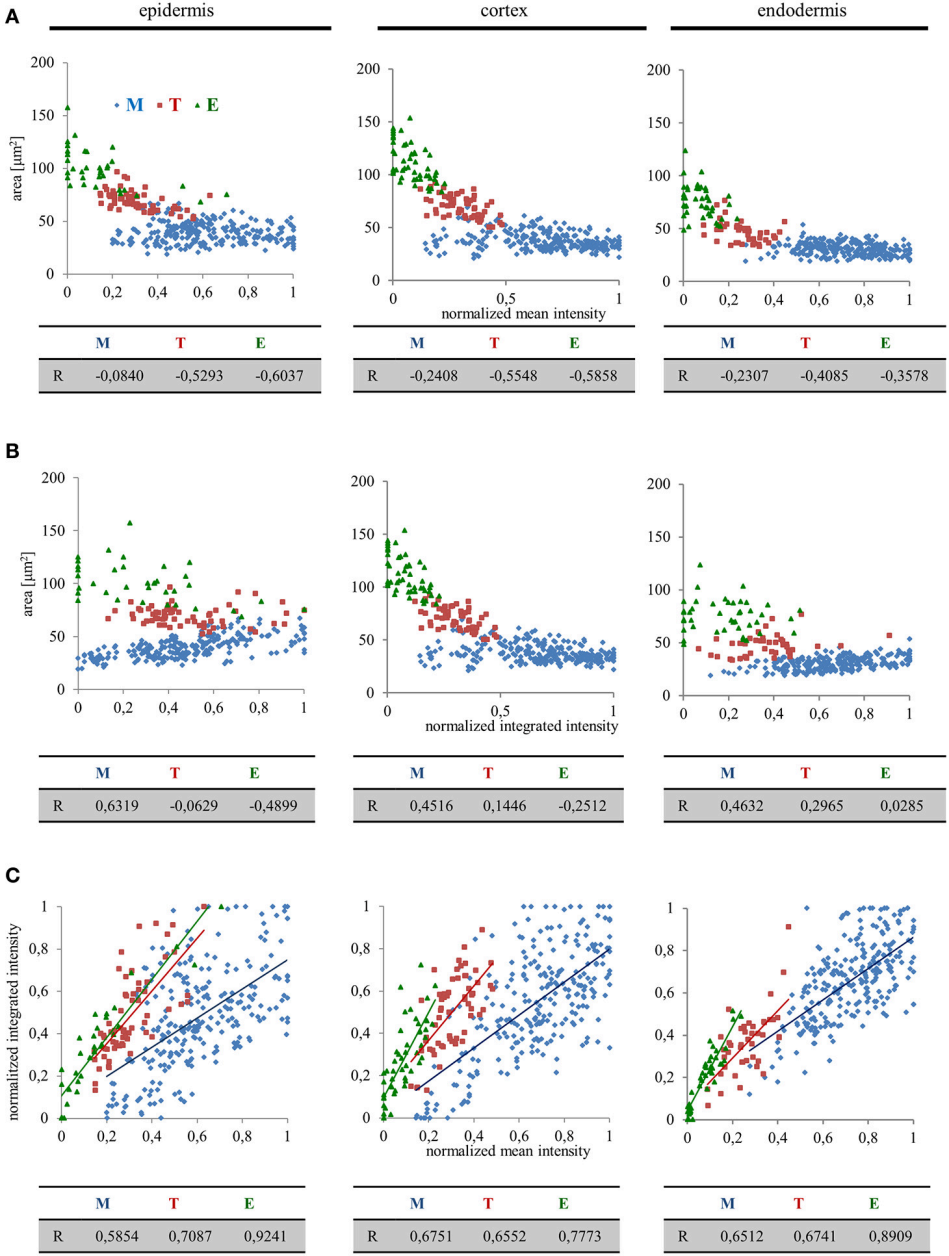
**Quantitative correlation analysis of nuclear surface area, nuclear EB1c-GFP mean signal intensity, and nuclear EB1c-GFP integrated signal intensity in diverse tissues and developmental root zones**. Separate graphs presenting correlation between nuclear surface area and nuclear EB1c-GFP mean signal intensity **(A)**, nuclear surface area and nuclear EB1c-GFP integrated signal intensity **(B)**, and nuclear EB1c-GFP mean signal intensity and nuclear EB1c-GFP integrated signal intensity **(C)**. Data from 4 individual cell files were collected and evaluated separately for epidermis, cortex and endodermis from two independent roots. Data are plotted separately for meristem (M, blue dots), transition zone (T, red dots), and elongation zone (E, green dots). Pearson's correlation coefficients (R) are shown for each individual cell developmental zone of the root apex.

From the data presented above it is apparent that there might be a high degree of correlation, positive or negative, between nuclear size and EB1c-GFP expression levels. We pursued this idea by quantitative correlation analysis plotting nuclear surface area, mean signal intensity (mean values per object area) and integrated signal intensity (sum values per object area) to clarify, whether the decrease of EB1c-GFP mean signal intensity during passage of root cells through cell developmental zones corresponds to the increase of nuclear surface area or not. This comparison was done separately for epidermal, cortical, and endodermal cells. In the meristematic zone, we observed the highest variability of the nuclear EB1c-GFP mean fluorescence signal intensity (Figures 3, 4) and thus not surprisingly, this zone exhibited the lowest correlation between nuclear surface area and nuclear EB1c-GFP mean signal intensity (Figure 6A). Measurements from transition zone and elongation zone were better correlated with prevalent negative values indicating that the decrease of EB1c-GFP mean signal intensity in the transition zone and the elongation zone corresponded mainly to increase of nuclear surface area and not to decline of EB1c-GFP expression levels. This suggests prior regulation of EB1c protein in the meristematic zone where cells are actively dividing. Most importantly, values from these three cell developmental zones were separated, albeit partially overlapping at their borders in the respective correlation scatter plots (Figure 6A). This observation proves the regular passage of cells from one developmental zone to another. Similar correlation analysis between nuclear surface area and nuclear EB1c-GFP integrated signal intensity revealed again the partial separation of measurements from meristematic zone, transition zone and elongation zone in all three measured tissue layers, although lower correlation coefficients and higher variability, especially in epidermis and endodermis, were apparent (Figure 6B). The highest intensity of EB1c-GFP fluorescence signal was measured in the meristematic zone, however, it was largely fluctuating (Figure 4). In accordance with this fact, the correlation between nuclear EB1c-GFP mean signal intensity and nuclear EB1c-GFP integrated signal intensity was lowest in the meristematic zone (Figure 6C). It could reflect cell cycle-dependent regulation of EB1c in the zone of mitotically-active root cells. In the transition zone and the elongation zone expression of EB1c-GFP decreased opposite to the increased nuclear area, observation which was corroborated by the higher positive correlation coefficients for comparing nuclear EB1c-GFP mean signal intensity and nuclear EB1c-GFP integrated signal intensity (Figure 6C).

## Discussion

### Development of environmental imaging in light-sheet microscope

The growing plant root system is physically challenged in its natural soil environment. Root growth is strongly affected by the mechanical properties of the soil causing direct changes in root cell length and width compared to experimentally grown plants (Bengough et al., 2006; Panteris et al., 2013). This may also indirectly affect the shoot growth (Jin et al., 2015). Conversely, in the conventional experimental setup, seedlings are mostly cultivated on air—solid medium interface which is significantly differing from the physical properties of the soil. Such a setting may cause the misinterpretation of the results, however, these cultivation methods are widely used and therefore widely accepted. Mimicking of the soil conditions in experimental environment is a challenging task (Okamoto et al., 2008). In the light-sheet microscopic setup, the seedling root is growing embedded in a solid agarose matrix and thus root growth is challenged by the rigidity of the medium, as happens during growth in the soil.

Another advantage of the light-sheet microscope is the ability to carry out long-term experiments at near-physiological conditions allowing root recordings with minimal stress (Maizel et al., 2011; Ovecka et al., 2015). By performing live cell imaging of 2-days old *Arabidopsis* seedlings for 2–5 h inside the light-sheet microscope, we demonstrate that by using light-sheet microscopy, plants grow in healthy conditions and thus they might be studied at the subcellular level with minimal limitations. Additionally, the light-sheet setup allowed for fast and up to some extent aberration free imaging at a considerable depth of the root, providing the opportunity to quantify nuclear levels of EB1-GFP not only for epidermis, but also for cortex and endodermis.

### Nuclear localization of EB1c

EB1c, the distinct subtype from EB1 family, is specifically present in vascular plants. It contains at its unique C-terminal sequence two composite motifs that serve as nuclear localization sequence (Komaki et al., 2010). Besides, our *in silico* search showed putative nuclear export signals at positions 213L, 215I, 217S, and 218L for EB1c. These findings suggest that EB1c is not tightly enclosed in the nucleus and that it may follow a canonical routine of active nucleocytoplasmic shuttling. Thus, EB1c cytoplasmic localization during mitosis and cytokinesis might be independent to nuclear envelope breakdown. The observation of EB1c localization in nuclei of post-mitotic, non-dividing cells such as those of the transition zone and the elongation zone, suggests that it might have a nuclear function. Thus, the visualization of nuclear EB1c-GFP in cells up to the differentiation zone suggests that EB1c might not be exclusively necessary for mitotic progression but rather have a broader role.

EB1c localization and function was previously documented in dividing cells of *Arabidopsis thaliana* (Dixit et al., 2006; Bisgrove et al., 2008; Komaki et al., 2010; Ho et al., 2011). In all the above studies, EB1c was studied in the context of cell division and studies were focused on the localization of EB1c at the microtubule plus ends with special emphasis on phragmoplast.

However, the evident interphase nuclear localization and function of EB1c was not systematically addressed in these former studies. For this reason we surveyed the nuclear occurrence of EB1c not only in the interphase cells of the meristematic zone, but also in post-mitotic non-dividing cells of the root transition and elongation zones. Measuring nuclear parameters revealed clear distinction among root developmental zones and correlated them with specific patterns of EB1c accumulation in the nuclei of different tissues and in both meristematic and post-meristematic root zones. Thus, EB1c-GFP can be considered a reliable physiological nuclear marker for root developmental studies including post-meristematic cells. With the help of previously published data about longitudinal root zonation (Dello Ioio et al., 2007; Baluška and Mancuso, 2013; Panteris et al., 2013), we identified particular zones in *Arabidopsis* plants expressing EB1c-GFP. As expected, EB1c-GFP signal was present in all nuclei across the studied root zones and tissues. We thus employed correlative quantitative studies monitoring developmental fluctuations in EB1c-GFP expression levels with the trend of nuclear size increase which is observed in the shoot-ward root growth gradient. Inversely to the nuclear area increase, expression of EB1c-GFP showed root-ward trend within all tissues along the longitudinal root axis with the highest intensity peak in the meristematic zone. The highest expression level and the lowest correlation between EB1c-GFP mean intensity and nuclear surface area in the meristematic zone are in accordance to previously published data about the role of EB1c in the cell division progression. *eb1c* mutants showed defects in spindle pole alignment, chromosomal segregation and phragmoplast orientation, however, the organization of the preprophase band was not impaired (Komaki et al., 2010). Nevertheless, EB1c might have a dual function in meristematic cells, depending on its subcellular localization (one on microtubule plus ends during mitosis and another one in nuclei during interphase).

More importantly we demonstrate for the first time, an evident persistent localization of EB1c in the nuclei of post-meristematic, non-dividing root cells residing within the transition and the elongation zones. As such this is the first study using quantitative advanced light-sheet microscopy to follow nuclear changes in correlation to the nuclear accumulation of a native cytoskeletal protein (EB1c was expressed under its native promoter) during development of the primary root. There seems to be a turning point for the expression of EB1c protein in the transition zone, where division is ceased while differentiation and endoreduplication are progressing. From this developmental point, expression of EB1c seems to be mechanistically related to increase in cell nucleus size which is carried out by endoreduplication. What exactly happens at the transition point and how switch from mitotic division to endoreduplication occurs is not well documented (del Pozo et al., 2006; Ishida et al., 2009, 2010; Adachi et al., 2011; Heyman et al., 2011; Doskocilova et al., 2013).

Our analyses highlight the nuclear localization of EB1c, opening in this way the hitherto unexplored field with several possible questions. According to its size (37 kDa) native EB1c is within the limits of passive diffusion through the nuclear pore (Brandizzi et al., 2012). However, we have never observed cytoplasmic localization of GFP-tagged EB1c in interphase cells suggesting that somehow it is tethered to the interphase nucleus. Whether this tethering is of functional significance or it serves storage purposes extends beyond the scope of the present manuscript. Nevertheless, it was of interest to see that EB1c is strongly predicted to interact with essential cell cycle check point proteins with nuclear localization during interphase. Using the STRING v10 protein interaction database (Szklarczyk et al., 2015) with a high stringency cutoff (0.9) we found putative interactors of EB1c, that include MAD2 (spindle assembly checkpoint protein with nuclear localization during interphase and kinetochore localization during mitosis; Ding et al., 2012), BUB3 (cell cycle arrest protein; Paganelli et al., 2015), and BUBR1 (checkpoint serine/threonine-protein kinase; Paganelli et al., 2015). Whether EB1c interacts with the above checkpoint proteins and/or other nuclear proteins and whether the nuclear presence of EB1c is of any functional significance are matters under investigation.

## Author contributions

DN and AK cloned constructs and transformed *Arabidopsis* plants. DN made immunoblot experiment. MO and DN performed live bioimaging using light-sheet microscopy. DN, MO, and AK performed image analysis and data evaluation. JŠ designed and coordinated all experiments and also participated on data interpretation. DN, AK, MO, GK, and JŠ wrote the manuscript. All authors approved the final manuscript.

### Conflict of interest statement

The authors declare that the research was conducted in the absence of any commercial or financial relationships that could be construed as a potential conflict of interest.

## Supplementary material

Supplementary Figure 1 **Predictions of putative nuclear export sequences in EB1a/EB1b (A) and EB1c (B) proteins using the NetNES 1.1 server (la Cour et al., 2004). (A)** Analysis of EB1a and EB1b proteins showed one putative NES at the position 193I in EB1a protein; however, the score is low over the threshold. Main domains are marked at the DOG protein model (Ren et al., 2009). **(B)** Analysis of EB1c protein showed four putative NES at the positions 213L, 215I, 217S, 218L. Main domains, D-motif and putative nuclear localization sequence are marked at the DOG protein model of EB1c. NN, neural network algorithm; HMM, Hidden Markov Model algorithm; NES score, combination of NN and HMM algorithms; CH, calponin homology domain; EBH, end binding homology domain; D, D domain (serves as a platform for interaction with MAPK).

Supplementary Movie 1 **Root growth of ***Arabidopsis thaliana*** seedling, expressing EB1c-GFP, observed by light-sheet microscopy**. Root of 2-days old plant was recorded for a time period of 5 h. Movie was produced from time-lapse image acquisition of every 2 min and is presented in the speed of 14 fps.
