# Supplementary material for: Developmental Nuclear Localization and Quantification of GFP-Tagged EB1c in Arabidopsis Root Using Light-Sheet Microscopy
Source: Front Plant Sci. 2016 Jan 5;6:1187. doi: 10.3389/fpls.2015.01187 (PMC4700127; doi:10.3389/fpls.2015.01187)
Supplement: Supplementary Figure 1 — Predictions of putative nuclear export sequences in EB1a/EB1b (A) and EB1c (B) proteins using the NetNES 1.1 server (la Cour et al., 2004). (A) Analysis of EB1a and EB1b proteins showed one putative NES at the position 193I in EB1a protein; however, the score is low over the threshold. Main domains are marked at the DOG protein model (Ren et al., 2009). (B) Analysis of EB1c protein showed four putative NES at the positions 213L, 215I, 217S, 218L. Main domains, D-motif and putative nuclear localization sequence are marked at the DOG protein model of EB1c. NN, neural network algorithm; HMM, Hidden Markov Model algorithm; NES score, combination of NN and HMM algorithms; CH, calponin homology domain; EBH, end binding homology domain; D, D domain (serves as a platform for interaction with MAPK). [file Presentation1.PDF]

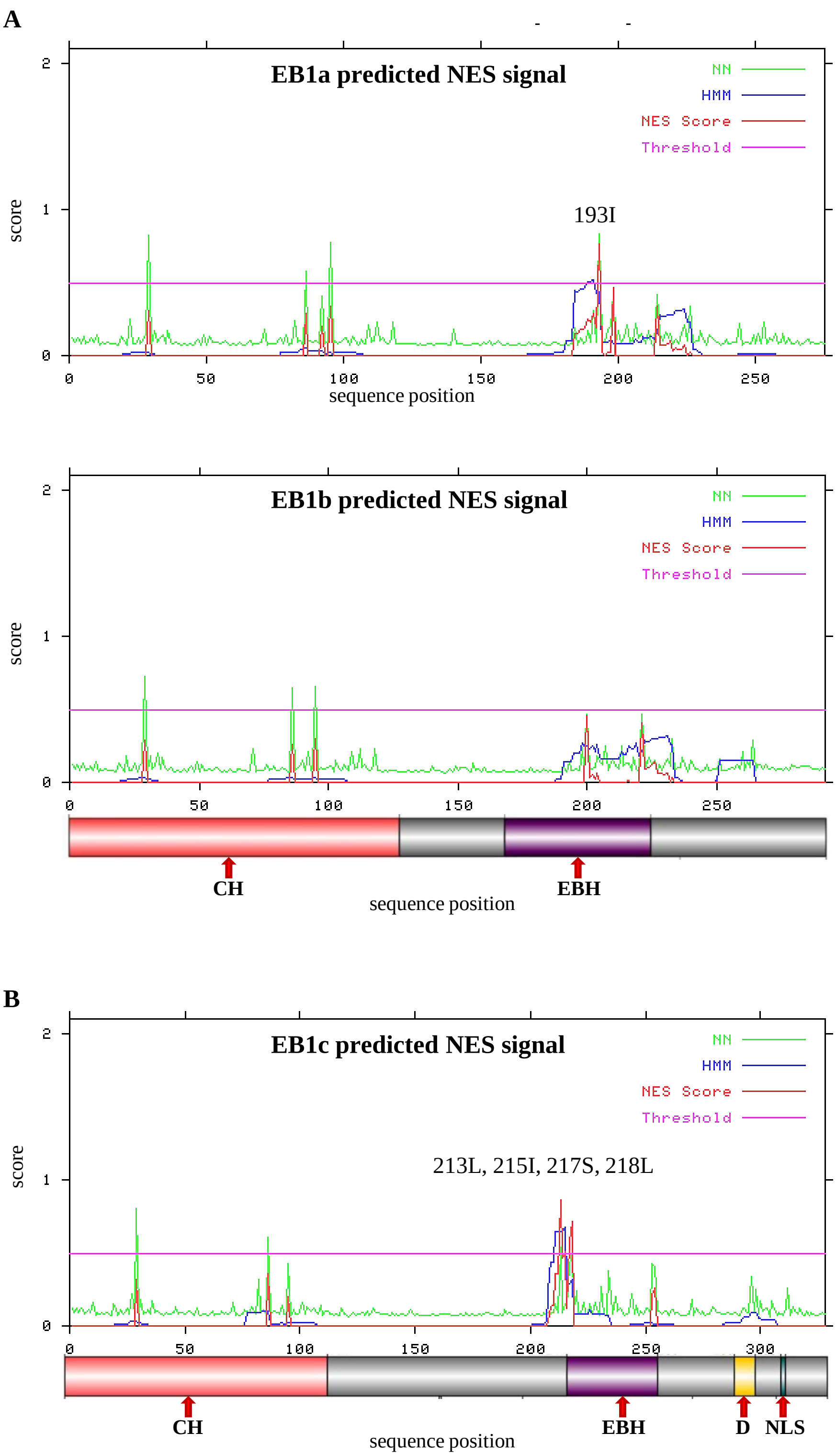

SUPPLEMENTARY FIGURE 1. Predictions of putative nuclear export sequences in EB1a/EB1b and EB1c proteins using the NetNES 1.1 server (La Cour, et al., 2004).
(**A**) Analysis of EB1a and EB1b proteins showed one putative NES at the position 193I in EB1a protein; however, the score is low over the treshold. Main domains are marked at the DOG protein model (http://dog.biocuckoo.org/). (**B**) Analysis of EB1c protein showed four putative NES at the positions 213L, 215I, 217S, 218L. Main domains, D-motif and putative NLS are marked at the DOG protein model of EB1c. NN - neural network algorithm; HMM: Hidden Markov Model algorithm; NES score: combination of NN and HMM algorithms; CH: calponin homology domain; EBH: end binding homology domain; D: D domain (serves as a platform for interaction with MAPK).
